# Towards a process management life-cycle model for graduation projects in computer engineering

**DOI:** 10.1371/journal.pone.0208012

**Published:** 2018-11-29

**Authors:** Murat Yilmaz, Faris Serdar Tasel, Ulas Gulec, Ugur Sopaoglu

**Affiliations:** 1 Department of Computer Engineering, Çankaya University, Ankara, Turkey; 2 HAVELSAN Inc., Ankara, Turkey; 3 Department of Computer Engineering, TOBB University of Economics and Technology, Ankara, Turkey; Universidade de Mogi das Cruzes, BRAZIL

## Abstract

Graduation projects play an important role in computer engineering careers in which students are expected to draw upon their knowledge and skills that were acquired since admission. To manage the activities of graduation projects, an iterative and incremental approach which aims continuous improvement is proposed as an alternative to a controversial delivery model. However, such integration brings up a set of challenges to be taken into account: e.g. multiple project deliveries, more labor-intensive effort from instructors, and ultimately continuous learning for all participants. One promising way to achieve such an integrated and continuous deployment velocity is to eliminate potential bottlenecks by giving student teams to receive early and continuous feedback. To this end, we propose a continuous feedback and delivery mechanism for managing the life-cycle of a graduation project through draft proposal, literature review, requirements gathering, design, implementation and testing which should produce intermediate outputs at predefined intervals. Most importantly, our approach makes it possible to quantify most of the activities involved in life-cycle process with various rubrics (i.e. measurement scales) that have been purposefully developed. The proposed model promotes the fact that all improvements should be monitored, evaluated and documented. The results of this study indicate that students who were managed using this approach produced better project deliverables and ultimately have delivered better and successful projects.

## Introduction

In the field of computer engineering, graduation project-based courses help students to develop their social (e.g. verbal and written expression skills) and technical (e.g. design and programming) skills, so that senior students might better benefit from the courses [1]. Through these practices, students have the opportunity to use much of the theoretical knowledge and practical experience they have gained in their educational life. For example, software, hardware or an embedded system project can be assigned as a comprehensive group project that includes requirements analysis, system design, implementation and acceptance testing. Starting with the concept design, all the way through to system analysis and software design, the students have opportunities to use the knowledge they have gained in different courses in a practical teamwork situation. In addition, detailed and comprehensive documentation practices are crucial for completing the projects, as recommended by other researchers [2]. As an initial part of the study, a literature survey needs to be conducted on the subject matter, which increases the breadth of knowledge of the students during the preliminary stages of work. The supervised students, based on academicians’ experiences, have contributed positively to the improvement of the process quality that ultimately affects the quality of the project’s delivery. To ensure that students understand the market and make a better transition to work-life, a number of seminars have been organized which are performed by a selected group of experts from the software industry.

Engineering education depends on the understanding of students’ perception and the development of teaching methods for engineering problems [3]. In common with all engineering disciplines, graduation projects are seen as an important part of the students’ training [4]. Particularly, in the engineering field, a graduation project work is one of the most important activities that students should undertake during their educational lives [5]. The universities that are considered in world university rankings, graduation projects emcompases all aspects of software development lifecycle, e.g. reqiurement anayisis, implementation and testing phases in accordance with computer industry practices [6].

Project-based learning (PBL) may be regarded as one of the practices that can bring students closer to real problems [7]. As a dynamic educational approach, PBL is a student-based pedagogical structure that renders the possibility of exploring real-world problems and finding answers to complex problems. This approach has been considered in order to prevent the student from being trained on a more crudely based or on an instructor-dependent basis [8]. According to Waycal [9], the selection and implementation phases of projects must be carefully applied, and valuable industrial projects must be produced considering that engineering graduation projects are the most comprehensive projects in the university life of seniour students. It has been observed that in the projects carried out in cooperation with industry, the students have taken an efficient role [10]. Although the PBL approach highlights students’ ability to work better, problems arising from flaws in workload distribution and disruption of the work undertaken by project team members have been seen as the weaknesses of this approach [11]. Students should also identify their deficiencies in general skills as well as technical skills and seek to collect information from academic sources and analyze this information, write reports and improve oral presentation skills. According to the Accreditation Board for Engineering and Technology, Inc. [12], engineering students should be tested for their ability to make technical presentations under the graduation project or senior project. A study [13] reports that one of the common problems in many academic education institutions with an international student quota is the problem of communication. Similarly, it has been reported that one of the frequent concerns emphasized in the studies on graduation projects is open communication and feedback problems [14]. In addition, another study [15] noted that the lack of *knowledge literacy* of industries could not be resolved. According to the American National Academy of Engineering, all engineering departments should endeavour to solve the inadequacy of individuals’ communication abilities and general skills in knowledge literacy and their related problems through their graduation projects in the direction of industry expectations [8]. The deficiencies of the general skills that are mentioned are as important as the lack of academic education in the outside world [16]. It has been pointed out that students are deprived of the ability to question something which is naturally expected from a young engineer. Moreover, inadequate student profiles in problem solving are frequently encountered [17, 18].

Although PBL has a lot of advantages such as improving collaboration, active participation, continuous learning, researchers report a drawback that students could have a tendency to ignore the projects in non-PLB courses given in the same semester [19]. In addition, there might be a variation regarding complexity of the projects that formed the project pool which is frequently created in PBL-based education approach [20]. In other words, good project are likely to teach more to its participants while a simple project may not offer the same learning outcomes. Consequently, it is more challenging for a PBL course to track what students learned in terms of the teaching standards. However, other than theoretical knowledge future employees are always interested in students who are more equipped with hands-on experience which can be reached by a PBL strategy.

Technological competence, in addition to the speed of access to information, is a capability that must be earned for graduation projects as it is one of the most important factors affecting the learning outcomes of students. In the light of evolving recent technologies, education systems require personalization of learning based-on known information technologies [21]. Although the use of technology alone does not guarantee learning, it is expected that the development of individuals’ abilities, such as creativity, communication and cooperation, will form milestones of the educational process structure that is to be constructed [22]. Hence, it would be easier to achieve the desired course output if students gained the aforementioned general skills prior to registering for the graduate project course.

In this article, a process management life-cycle model will be introduced to facilitate the completion of the graduation project in CENG 407 Innovative System Design and Development I and CENG 408 Innovative System Design and Development II, as well as facilitating the completion of two semesters in the education curriculum of Çankaya University Computer Engineering. The remaining part of this paper is organized in the following manner. First, it contextualizes the research by providing background information about project-based learning from an engineering perspective. Next, materials and methods section explains the implementation process in detail. In the third section, the results obtained from the management process is discussed using a set of specific methods by which the research and analyses were conducted. Finally, the article will conclude with some thoughts on implications and findings.

## Materials and methods

### Graduation projects process management infrastructure

This part of the work introduces a process life-cycle model developed within the scope of improving the control and implementation activities of the Çankaya University Computer Engineering department graduation projects. In addition, the effects of the applied learning and teaching approaches on the structured process and on the recurring improvement activities will be discussed.

The goal here is to contribute positively to graduation project students’ ability to write, speak and present in society in order to gain the “general communication” abilities. For this purpose, students are expected to report on their work and in accordance with the technical procedures they had taken. In the first stages of the graduation project, the students are required to complete a document which contains information about their literature reviews as an intermediate output. With the help of this output, the technical documentation skills of the students have begun to be improved even in the preliminary stages of the process. These improvements are activities that are expected to be given importance by European and American engineering education and accreditation institutions [23, 24]. To support the whole managerial process, the seminars are conducted by industrial experts. In particular, experts specializing in novel topics in the computer engineering domain are brought together with the students.

This study is being conducted as part of accreditation requirements and it is based on a set of course projects and, thus, not considered *human* subjects *research* by definition. The protocol that we have used to conduct this study would not constitute a human subject research based-on human subject office at the University of IOWA (http://hso.research.uiowa.edu/studies-are-not-human-subjects-research). Firstly, the data is collected just for internal improvements and educational purposes from a set of class exercises, assignment, and a set of feedback sessions that were not intended for use outside of the classroom. Secondly, conducted surveys were for administrative purposes (e.g. teaching evaluations), Thirdly, the goal of the survey was not collecting identifiable private information of participants but to improve a service. In light of this remarks, this study is not considered as a human-subject research and therefore it does not necessarily require an approval from ethics committee. Consequently, this study was not found within the scope of the *ÇANKAYA University Ethics Committee* and it has not been reviewed by research ethics committee. All students and instructors gave their informed consent and volunteered to participate in the study, all of whom were made aware of the aim and procedures. They were informed verbally in all data collection processes.

Computer engineering graduation projects at Çankaya University last for two semesters. In the first semester, students are asked to select a project, research the area they have selected to improve themselves, search the literature, close the missing, and report on all their work. In the second term studies, it is expected that they should carry out product development, product acceptance and user tests and report on all these studies.

**Fig 1:**
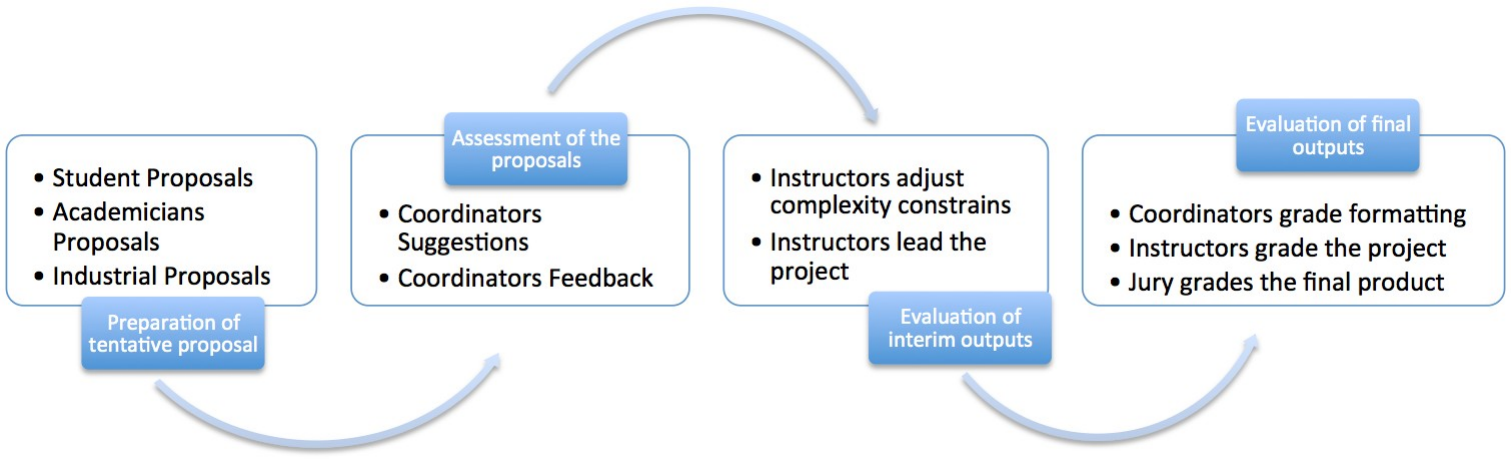
Graduation projects process management model.

The graduation project management process of the projects that we prepared is expressed as four main sub-processes, as shown in Fig 1.

1. Preparation of tentative proposals: As Association for Evaluation and Accreditation of Engineering Programs (MUDEK) points out, an important issue can be characterized as the need to increase complexity in graduation projects. In order to solve this problem, more project suppliers are especially considered. In particular, instructors and students acquire projects from companies as previously applied. The instructors are also able to propose projects in line with their work areas. It is also possible for students to propose projects based on their interests in order to increase their motivation.
2. Assessment of project proposals: At this stage, projects from all providers are standardized with the help of a project proposal form. Precautions should be taken to ensure that proposals be received at the beginning of the period. Graduation project proposals are checked by the project coordination committee for complexity levels, predetermined constraints and potential risks, and then valid project proposals are identified by eliminating ineligible proposals. However, coordinators may ask for a revision proposal from its owner, if necessary, to improve the complexity of the project, potential risks, and project definition. In this way, proposals that were originally marked with missing points might be reconsidered.
3. Evaluation of interim outputs: In this phase, students are asked to participate in a literature survey, write the SRS and SDD documents along with the main outputs expected from the project teams. These outputs contain intermediate stages that are updated throughout the period and are effectively used to form the project term report. In addition, a pre-evaluation study is provided by supervising the conformity of these outputs with academic norms (e.g. order of text, naming of forms and tables and referencing correctly, etc.) by graduation project coordinators. Thus, the difficulties that students may experience in the reporting process may be addressed in advance thereby enabling the project supervisor to focus more easily on the project.
4. Evaluation of the final outputs of the project: Graduation projects are evaluated by the teaching staff conducting academic counselling at the end of the semester. This evaluation process is conducted with the aid of the literature, the SRS and SDD evaluation scales established by the coordinator. Through such improvements, it is possible to introduce a certain standard in the grading process.

### Graduation projects management plan

This plan is designed to show the road map to be followed by students, and instructors (i.e. academic advisors) of students and CENG 407/408 project coordinators in the projects to be carried out under the CENG 407 and 408 graduation projects. Throughout the study, a semester is accepted as 14 weeks.

**Fig 2:**
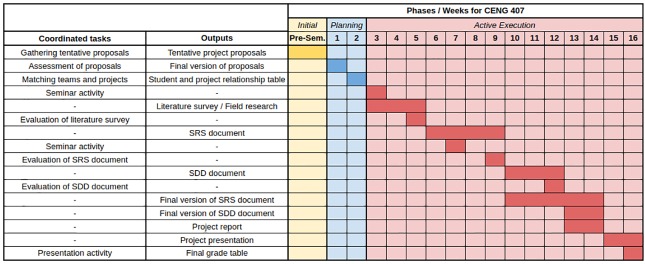
Gantt chart for CENG 407.

Fig 2 illustrates a Gantt chart for CENG 407 course respectively in which the tasks undertaken by the project coordinators and desired outputs are matched to the corresponding phases and weeks in an academic semester. Note that evaluation tasks indicate the grading processes concerning project coordinators which determine the partial grade of each documented item.

#### CENG 407 Projects

The period of 14 weeks in one semester is determined as follows:

Week before the beginning of the semester: Project Proposal Forms must be submitted to the project coordinator by the beginning of the first week of the semester. The text of the draft project proposal should be submitted to the project coordinator by the end of the first week by the departmental faculty members. The draft should be at least 150 words. It should contain the essence and significance of the project. The project may be based on the company or it may be proposed by instructors. The instructor recommending the project may expand and elaborate on the scope of the project if it is deemed necessary by the company (if the project is not complicated). The coordinator is authorized to inform the proposer of the project if the standards are not followed and improvements are required to the draft.

**Week 1:** Draft proposals that are to be delivered at the beginning of the first week are evaluated by the coordinator. If necessary, the coordinator will return them for revision. Projects are announced to students after the proposals are finalized. In addition, the management plan of the graduation project is announced to students through a joint meeting to be held. This week’s interim output is the summary of the project draft.

**Week 2:** By the end of this week, students are asked to create a project group (of 3 or 4 students depending on the project) and apply to the projects they desire. This week’s interim output is the student and project relationship table. Groups should be decided by the end of the period of the add-drop. Project selection forms are approved by students, departmental members and the department chair. The students are informed by their advisors that they should prepare a literature review and/or conduct a detailed field survey on the subject in the following three weeks and prepare a report of at least 1000 words about the project documents they have already prepared.

**Week 3:** This week, detailed report preparation studies on the literature review are started. During this week, group members work together with their advisor by meeting once per week. Each meeting is recorded on a project tracking form. Two copies are kept for the advisor and for the student. This week, a seminar is held on “Lifelong Learning Needs, Access to Information, Monitoring of Developments in Science and Technology” to inform the students. Student participation is compulsory.

**Week 4:** Study of the literature review continues. This week, a presentation is made on the basics of SRS and SDD preparation to inform students. Student participation is compulsory.

**Week 5:** Literature surveys or field research reports from project groups are submitted. Hence, this week’s interim outputs will consist of literature reviews and field research reports. The literature review should begin with a 150-word abstract. The abstract should be written in both Turkish and English. The main text of the report should be at least 1000 words and should be supported with references. References use the IEEE citation and writing style. The evaluation of this document is conducted according to the evaluation rubric determined for the literature review.

**Week 6:** Project groups start studying for the necessary examination, interview and evaluation in order to prepare the SRS document.

**Week 7:** Students continue to study for the SRS. This week, a seminar is held on current topics to inform students. Student participation is compulsory.

**Week 8:** Students continue to study for the SRS.

**Week 9:** At the end of the week, the printed copy of the SRS is delivered to the project coordinator. In addition, an electronic copy of the SRS is to be uploaded to the Graduation Project Repository (GPR). The evaluation of the document occurs according to the evaluation rubric determined prior to the SRS. This week’s interim output is the SRS document.

**Week 10:** Project groups start the necessary examination, meeting and evaluation work in order to prepare the SDD.

**Week 11:** Students continue to study for the SDD.

**Week 12:** This week, a printed copy of the SDD is delivered to project coordinator. Additionally, an electronic copy of the SDD is uploaded to the GPR. The evaluation of the document is conducted according to the evaluation rubric determined prior to the SDD. This week’s interim output is the SDD.

**Week 13:** Preparation for the semester report commences.

**Week 14:** Project reports for all projects are to be prepared such that they include a literature review, the SRS and SDD in terms of content. An electronic copy of the report is uploaded to the GPR and a printed copy of the report is to be delivered to the project coordinator. Outputs of the week consist of a project report and final versions of the SRS and SDD.

**Week 15:** It is expected that the presentation document be uploaded to the GPR. Therefore, the presentation document is accepted as the output of the week. This week, project groups present their projects pursuant to a project presentation program to be prepared by the coordinator. The presentation is evaluated by using the presentation evaluation rubric for the course (CENG 407). The project outputs are evaluated by the advisor with the pre-determined evaluation rubric and they are delivered to the project coordinator with the evaluation results signed. The projects are graded.

To balance the effect of coordinator(s), instructor(s) and the jury on project outputs, a collaborative grading approach was constructed. The grading ratio were based on a set of discussion held by academic board of the department including but not limited to head of department, graduation project coordinators, and instructors (i.e. academic advisors). In this grading model, project output and literature review are collaboratively graded by both instructors and graduation project coordinators. The goal is to monitor and report any problems of the process in early stages. The board suggests a limited amount of grading which needs to be given to coordinators so that they can manage students more easily.

The projects are marked according to the scores given in Table 1. Advisors are the instructors who are managing the student’s projects. Jury is a group of instructors who are grading the project progress and students’ presentations.

**Table 1:** CENG 407 project outputs and scores.

Project Output	Evaluator and Effect	Score
Literature Review	Coordinator (25%) and Advisor (75%)	10
SRS	Coordinator (25%) and Advisor (75%)	15
SDD	Coordinator (25%) and Advisor (75%)	15
Final Version of SRS	Advisor (100%)	5
Final Version of SDD	Advisor (100%)	5
Project Report	Advisor (100%)	15
Ethics and Social Responsibility	Advisor (100%)	5
Project Presentation and Jury Evaluation	Jury(100%)	30
TOTAL		100

During the evaluation process, the coordinator only evaluates the literature review, SRS and SDD. Part of the score given by the project coordinator to students does not exceed 25% of the total score of the first three outputs (contribution of the coordinator to the total score is 10/100). The total grade taken from the outputs is translated into a letter grade with the help of the table given in Article 25(5) of the Associate Degree, Undergraduate and Instruction Regulation of Çankaya University. (http://kutuphane.cankaya.edu.tr/wp-content/uploads/sites/42/2018/05/013_1.docx).

#### CENG 408 projects

**Fig 3:**
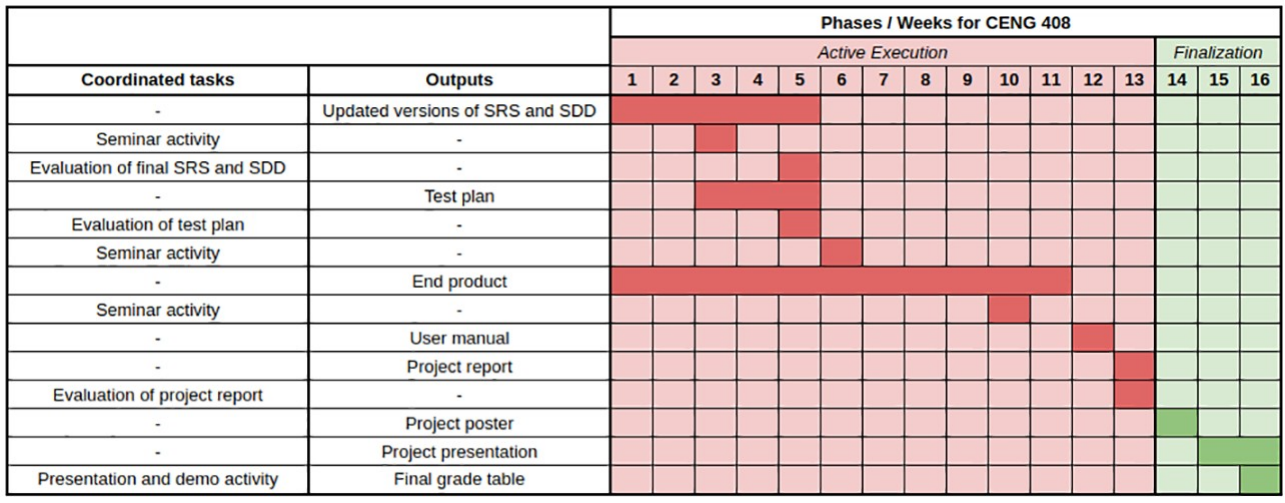
Gantt chart for CENG 408.

Fig 3 depicts a Gantt chart for CENG 408 course respectively in which the tasks undertaken by the project coordinators and desired outputs are matched to the corresponding phases and weeks in an academic semester. Note that evaluation tasks indicate the grading processes concerning project coordinators which determine the partial grade of each documented item.

The 14 week period of a semester is specified as follows:

**Week 1:** Study of updating the SRS and SDD and the implementation process commences.

**Week 2:** Study of updating the SRS and SDD and the implementation continues.

**Week 3:** Test plan preparation studies are started and the application development process continues. This week, a seminar on “Entrepreneurship, Innovation and Sustainability” is held to inform the students. Student participation is compulsory.

**Week 4:** Test plan preparation studies are started and the application development process continues.

**Week 5:** At the end of the week, printed copies of the updated SRS and SDD are delivered to the project coordinator by project groups, and electronic copies of these documents are uploaded to the GPR. The evaluation of the documents occurs with the help of the evaluation rubric for the SRS and SDD. This week’s interim outputs consist of updated SRS, SDD and test design documents.

**Week 6:** Implementation phase is continued. This week, a seminar is held on “Software Development Challenges” to inform students. Student participation is compulsory.

**Week 7:** Implementation phase continues.

**Week 8:** Implementation phase continues.

**Week 9:** Implementation phase continues.

**Week 10:** Implementation phase continues. Application tests are performed. The preparation process of the term project report commences. This week, a seminar is held on the current issue to inform the students. Student participation is compulsory.

**Week 11:** Application development process is completed. The source codes and compiled version of the project are uploaded to the GPR. This week’s output is the end product realized within the scope of the project.

**Week 12:** The user manual of the product is prepared, uploaded to the GPR and accepted as this week’s output.

**Week 13:** The term project report is to be completed by this week. An electronic copy of the report is to be uploaded to the GPR and a printed copy of it is delivered to the project coordinator. This week’s output is the project report.

**Week 14:** The project poster is prepared and an electronic copy of it is uploaded to the GPR.

**Week 15:** It is expected that the presentation document be uploaded to the GPR system. This week’s output is the presentation document. This week, the project groups will present their projects in accordance with the presentation program to be prepared by the coordinator. The presentation documents should contain details such as the project description, suggested solutions, the software development process and the product demo. Evaluation of the presentation occurs with the assistance of the evaluation rubric specified previously. The project documents and the produced product are evaluated by the advisor of the project and the evaluation result is submitted to the project coordinator as signed. Projects are marked using the scores specified Table 2. Advisors are the instructors who are managing the student’s projects. Jury is a group of instructors who are grading the project progress and final product(s).

**Table 2:** CENG 407 project outputs and scores.

Project Output	Evaluator and Effect	Score
Updated SRS and SDD	Coordinator (25%) and Advisor (75%)	10
Test Plan	Coordinator (25%) and Advisor (75%)	10
Project Report	Coordinator (25%) and Advisor (75%)	10
Final Product	Jury (100%)	40
Project Presentation	Jury (100%)	30
TOTAL		100

During the evaluation, the coordinator will only assess the updated SRS and SDD documents, the test plan and the project report. Part of the score given by the project coordinator to students will not exceed 25% of the total score of the first three outputs (contribution of the coordinator to the total score is 7.5/100). The total score taken from the outputs is translated into a letter grade with assistance from the table given in the related regulation.

## Results and discussion

To evaluate the implications of this study, a group of 83 people consisting of fourth year students of the Computer Engineering department who have enrolled in the CENG 407 and CENG 408 courses in the 2014-2015 and the 2015-2016 academic years were formed. Individuals who were taken these courses in the 2014-2015 academic years were not affected from the proposed approach. 33 of these 83 students completed their graduation project, CENG 407 and CENG 408 courses in the 2014-2015 academic year, while the remaining 50 completed their courses in the 2015-2016 academic year. The students’ thoughts about the lessons were recorded with the questionnaires that were prepared based on the learning outputs enforced by the Bologna Process in the course definition prior to the presentations of the projects made after the end of each term. Within the scope of these surveys, students were asked the following questions for the evaluation of the courses.

1. Did this course give you the ability to design and implement an innovative and fully functional end product?
2. Did this course gain for you the ability to practice system testing and its development phases?
3. Did this course give you the ability to write a detailed report about your project?
4. Did this course give you the ability to present your project in front of a jury?
5. Did this course give you the ability to use the necessary software and hardware tools?
6. Did this course give you the ability to work in teams as well as individually?
7. Did this course help you to gain the awareness of ethical and legal issues in the computer world?

In response to each of the above questions, students will select one of the following answers: *Strongly Agree, Agree, Neither Agree Nor Disagree, Disagree and Strongly Disagree*. In order to be able to perform a numerical analysis, the answers are numbered with the option *Strongly Agree* as 4, *Agree* as 3, *Neither Agree Nor Disagree* as 2, *Disagree* as 1 and *Strongly Disagree* as 0.

The questionnaire with 33 students who took related courses in the 2014-2015 academic year was administered on 3 June 2015 at the Çankaya University Central Campus Blue Seminar Hall. The mean scores out of 4 and standard deviations of each question in the questionnaire are given in Table 3.

**Table 3:** Results of survey made in the 2014-2015 academic year.

	Q1	Q2	Q3	Q4	Q5	Q6	Q7	OVERALL
**Mean**	3,36	3,18	3,15	3,21	3,24	3,24	3,24	3,23
**Std. Dev**	0,93	1,07	1,12	1,08	1,03	1,12	1,09	1,05

The survey with 50 students taking related courses in the 2015-2016 academic year was held on June 1, 2016 at Çankaya University Central Campus Auditorium 6. The mean scores out of 4 and standard deviations of each question in the questionnaire are given in Table 4.

**Table 4:** Results of survey made in the 2015-2016 academic year.

	Q1	Q2	Q3	Q4	Q5	Q6	Q7	OVERALL
**Mean**	3,38	3,74	3,62	3,28	3,40	3,38	3,32	3,45
**Std. Dev**	1,10	0,96	1,01	1,18	1,07	1,12	1,00	1,07

According to these results, it is seen that the results of the questionnaire given in the 2015-2016 academic year show higher averages both in the questions and in general. To obtain this result statistically, the questionnaire results were analyzed using two sample t-tests (using the Satterthwaite estimator variant) used for groups of different sizes. As a result of this statistical analysis, it was found that the averages obtained for Questions 2 and 3 increased significantly in the results of the questionnaire given in the 2015-2016 academic year compared to the 2014-2015 academic year. This result shows that the ability of students to develop and test their own systems as well as their ability to document of system developments has increased.

In addition to the students’ evaluation, educational objectives that were defined in the Bologna forms of the CENG 407 and 408 (see Table 5) courses were assessed by MUDEK accreditation committee. The goal is to explore how students’ tasks were performed during the measurement activities of the course such as quizzes, projects, etc. The MUDEK accreditation committee checked the score obtained by calculating the average of the grades of the students in the relevant tasks of the courses with respect to the educational objective in order to explore how successful the educational objective was fulfilled.

**Table 5:** Educational objectives of CENG 407 and 408 courses.

Educational Objectives (EO)
1. Ability to complete and apply information using limited or missing data; ability to integrate multidisciplinary information in one part.
2. Ability to develop original ideas and methods and ability to develop innovative solutions when designing systems, components and processes.
3. Ability to communicate both orally and in writing at the level of the European language portfolio B2 (English in particular).
4. Ability to explain the work progress and results clearly and in a systematic manner both verbally and in writing in national and international media.
5. Ability to describe environmental and social aspects of engineering applications.

Table 6 illustrates the overall average scores of students based on the educational objectives of CENG 407 course in both 2014-2015 and 2015-2016 academic years while Table 7 shows the scores of students with respect to the educational objectives of CENG 408 course in both 2014-2015 and 2015-2016 academic years.

**Table 6:** Average scores of the students in CENG 407 course.

CENG 407	EO1	EO2	EO3	EO4	EO5
**2014-2015**	78	67	90	86	82
**2015-2016**	91	86	90	92	88

**Table 7:** Average scores of the students in CENG 408 course.

CENG 407	EO1	EO2	EO3	EO4	EO5
**2014-2015**	87	82	83	84	84
**2015-2016**	90	88	88	90	88

According to the results in Table 7, students who took the CENG 407 course in the 2015-2016 academic year were more successful than the students who took the course in the academic year 2014-2015 in terms of educational objectives except the third educational objective. Accordingly, students who took the CENG 408 course in the 2015-2016 academic year were more successful than the students who took the course in the academic year 2014-2015 in all educational objectives. As a result, the educational objectives of the CENG 407 and 408 courses were fulfilled better in 2015-2016 academic year.

In addition to this quantitative analysis, a set of systematic interviews were conducted with the students (N = 10) of degree-taking projects in both the 2014-2015 academic year and the 2015-2016 academic year in order to increase the reliability of the work. The aim of this part is figure out the students’ assessments and experiments about the process applied in the graduation project. The snowball method has been used to complete the qualitative analysis. First of all, the students who completed the project in the 2014-2015 academic year were asked the following questions in order to obtain their views on this subject as a result of the graduation projects being managed in such a process.

1. Do you think your project would progress more regularly if the tasks given in the graduation project would be carried out step by step within a certain period of time?
2. If the project tasks were given step by step, do you think you can use time more effectively?
3. If a sub-product emerges after each step of the process, do you think this product would lead to the next step?
4. If your project has progressed step by step within certain time periods, did it encourage you to work harder?
5. If your project were managed by a process that consisted of the exact steps of the project at the beginning of the project and the exact dates that showed the due dates of each step, do you think this process would further improve the quality of the product you would have developed in your project?
6. If you wanted to report every step you developed in your project periodically, do you think it would improve your ability to produce more documentation?
7. If your projects were assessed with specific measurement parameters, would you consider the project evaluation process to be a standard evaluation?

One student mentioned:

**Interview quotation:**“If the tasks given in the graduation project were carried out step by step within a certain period of time, the project progressed more regularly and the students were directed more effectively about the project. Students will be able to use time more effectively if they are asked to do a given task within a certain period of time. In addition, if a sub-product of the final product emerged as a result of every step of the project, this process would be a much better way by both increasing the motivation of the students and by observing how the project was going. At the same time, this situation encourages the student to work harder and ensures that the final product is of better quality. If the project developments are reported at specific time intervals, it becomes easier for students to see any mistakes made by increasing their reporting abilities.”

As another interviewee explained:

**Interview quotation:**“I think managing the project with a systematic model will help students to notice the errors in the project more quickly. Thus, students can use time more efficiently and produce better quality products. Moreover, the expectation of producing sub-products at every step from the students leads the student to work harder and makes the product that will be released at the end of the project more useful. Documentation of each step will increase the ability of the student to make the project more understandable by other people.”

As another student expressed:

**Interview quotation:**“When the project does not progress step by step and tasks arrive irregularly, the project enters a very complicated situation. If the tasks progress step by step, students can use their time better. The production of a product at the end of each step will make it easier to understand the faults before developing the final product. Thus, students were better motivated to work and the final product would be of much better quality.”

Another participant declared that:

**Interview quotation:**“I think that projects would progress more regularly if the tasks given in the graduation project were done step by step within a certain period of time, and this also enabled us to use time effectively. The step-by-step progress of the project would motivate us, and the emergence of the product as a result of each step could lead us to a more accurate picture of the progress of the project. I think that every step of the processes would have a direct impact on the quality of the product if the requirements to do them and the timing are obvious. Apart from that, I believe that taking much time to report reduces motivation. In addition, it is important to set the standard for grading students to solve the inequality.”

As another student commented:

**Interview quotation:**“The assignment of tasks within a certain period of time would allow the student to gain a disciplined working habit, which meant that the project would progress more regularly. He could teach us the concept of time management which is very important today. In applications developed with an agile methodology, it would give us an idea about the next steps. Step-by-step and regular progress would be an extra motivation for the project. I am not sure that this type of development methodology increases the quality of projects. I think that the documentation phase improves us on reporting and I consider it important that these reports are evaluated by setting specific standards.”

To increase the reliability and validity of the results obtained from the qualitative analysis, expert reviews were conducted to assess students feedback. Five instructors were selected with at least 10 years’ experience on this topic evaluated the answers given by students during the interview. The evaluation of the questionnaire administered to the students who participated in the graduation project in the 2014-2015 education year is shown in Table 8.

**Table 8:** Evaluation of the experienced instrcutors.

	Interview 1	Interview 2	Interview 3	Interview 4	Interview 5
**Evaluator 1**	Positive	Positive	Positive	Positive	Positive
**Evaluator 2**	Positive	Positive	Positive	Positive	Positive
**Evaluator 3**	Positive	Positive	Positive	Positive	Positive
**Evaluator 4**	Positive	Positive	Positive	Positive	Positive
**Evaluator 5**	Positive	Positive	Positive	Positive	Positive

As a second step of the quantitative analysis, the following questions were asked to the students who completed the project in the 2015-2016 academic year in order to obtain their opinions about the process that was developed based on the results obtained from the first step.

1. Did the step-by-step tasks of the graduation project within a certain period help the project to progress more regularly?
2. Has the step-by-step assignment of project tasks increased your ability to use time effectively?
3. Did the product emerging after each step lead to the next step of your project?
4. Did the step-by-step progress of the project encourage you to work harder?
5. Did the process applied within the scope of the project increase the quality of the product you developed?
6. Has the process used in the project developed your ability to carry out documentation?
7. Have the measurement parameters used in the project evaluation provided a specific standard for the project evaluation process?

One participant reported:

**Interview quotation:**“Dividing the project into the modules and sequentially progressing in the project has helped to identify short-term targets and to better analyze the work in the end. Weekly job descriptions provided regular reviews and evaluations of the project. The emergence of a product after each iteration allowed for the assessment of the applied changes and the manner in which the project would be followed. This has been an incentive for us to show our progress in the project and to work. Regular follow-ups of this applied process helps to eliminate and correct deficiencies in time. This situation leads to improvements in the quality of the product. Even though there are many positive thoughts about the process, due to the unclear situations about the documentation process, occasional wrong documents have been created, thus causing both loss of motivation and time.”

As another interviewee stated:

**Interview quotation:**“The step-by-step execution of tasks within a given period of time within the scope of the graduation project has enabled the project to be carried out on a regular basis, as is the case in many projects in business life. Such a time constraint has supported us to keep our performance and potential high. The assignment of tasks in a certain order made the project to be completed regularly and display our talents. As a result, we created a sub-product as a result of each step and used this sub-product as input for the next step. This situation prevented the complexities that could occur in the project by not allowing us to leave all the work to the last day. Being given detailed duties in the project allowed us to work on the project continuously. Consequently, this process has improved our ability to produce a quality product at the end of the project and to make any documentation related to the development of the product at every stage.”

As another student commented:

**Interview quotation:**“The step-by-step performance of the tasks given within the graduation project has helped the project to progress more regularly, however, in some cases, when the due dates of the task were coming closer, we did not focus on the project because we couldn’t manage the stress. We did not use the time effectively, so the other part of the job was affected. Although we produced a successful product at the end of the project, this process was difficult for us.”

As one student stated:

**Interview quotation:**“The step-by-step construction of the project within a certain period of time allowed us to see the deficiencies and redundancies of the project. Thus, this contributed to the progression of the system in a systematic and orderly manner. The step-by-step assignment of tasks in the project brought with it regular work and enabled us to use our performance and time in the best possible way. This was guided for the next step since the product that emerged after each step was used in the next step. The time limit for producing the product at each stage has encouraged us to work with superior performance. Naturally, as a result of hard work, a successful product developed at the end of the project. In addition, since we regularly report every development on the project, our ability to report has greatly increased.”

Another participant declared that:

**Interview quotation:**“The step-by-step and planned execution of the project tasks over a period of time allowed the project to proceed progressively and successfully. Step-by-step tasks have made it easier for us who did not yet know the project management and processes, and this process, which is actually very complicated, has allowed us to use the time we use for our work more efficiently and productively. Through the sub-products obtained at each stage of the project development, we saw better what we needed to do next. Thanks to this process, we worked hard without losing our dynamism in order to be able to get these breakthroughs out and finally we could produce a successful product. As a negative thought, the project evaluation process has us going through negative criticism as a motivational dampener instead of positive reviews, which will improve us a lot.”

The results obtained in the second phase are shown in Table 9, which are identical to in the results of first stage evaluation with the same instructors.

**Table 9:** Evaluation of the experienced instructors.

	Interview 1	Interview 2	Interview 3	Interview 4	Interview 5
**Evaluator 1**	Positive	Positive	Negative	Positive	Positive
**Evaluator 2**	Positive	Positive	Negative	Positive	Positive
**Evaluator 3**	Neutral	Positive	Neutral	Positive	Neutral
**Evaluator 4**	Positive	Positive	Negative	Positive	Neutral
**Evaluator 5**	Positive	Positive	Neutral	Positive	Positive

When the results of the evaluations were analyzed, students, who developed their graduation projects in the 2014-2015 academic year and did not apply the newly developed project management model, thought that such a process would be very useful and positive. The students who had completed their projects in the 2015-2016 academic year and developed their projects within this management model gave generally positive feedback about the process.

This study set out with the aim to design a holistic approach for managing graduation projects in Computer Engineering which are considered as a cornerstone for students’ professional careers. The results of this study indicate that creating iterative chunks of tasks for measuring and monitoring graduation projects make the outcomes more manageable. The findings further support the idea that participants are happy to confront real-world like problems who needed to collaborate to create solutions, and ultimately present their results which improves both their social and technical skills concurrently. These results are likely to be observed by the evaluators who found the idea of continuous feedback and deployment encouraging based on a modularized structure of the process. This combination of findings provides some support for the idea of continuous management. The shared workload between the coordinators and instructors provide an efficient way to monitor and evaluate students’ projects. Our results shall serve as a practical reference for computer engineering departments to design, structure and supervise their projects. For example, we encourage instructors to make connections between project topics they would offer and align them with students’ personal interests. Secondly, we suggest that projects should be connected with industrial requirements rather than being a pet project. It was observed that many students are motivated when they are working on apart of a real and therefore an industrial problem.

## Conclusion

This study has shown that there has been an appreciable increase in the students’ ability to develop project proposals, project implementation, empirical work and communication when the proposed process management model for graduation projects is applied. To support this idea, a set of semi-structural interviews have been conducted with students who have done graduation projects both using this methodology and without using this methodology. According to the results of these surveys, all students who have completed the graduation projects in the 2014-2015 academic year without using this methodology have made positive feedback about the use of such methodology in graduation projects considering that they are aware of the importance of the project management process due to their relatively high work experience. Even though there are some negative thoughts of the students who have completed the graduation projects in the 2015-2016 academic year with using this methodology as the model pushes the students to work more intensively and harder, the students generally have positive thoughts about the management model.

The proposed managerial model for computer engineering graduation projects aims to establish a concrete structure for project life cycle which promotes the development of standardization in graduation projects by using a common framework. It aims to establish a set of processes for managing the graduation projects. Although the study has successfully demonstrated that it is benefical for managing the graduation project life-cycle, it has certain limitations. The present investigation has only considered the graduation project management therefore project teams should improve their software development processes by identifying project risks and critical problems that is depdendent to their product improvements. The proposed model is similar to ISO/IEC 12207 which does not prescribe a process model to follow but proposes a high level process architecture [25]. There are distinctive kind of graduation projects (e.g. web based-projects, virtual reality application, computational projects in image processing, mobile applications) which can be managed by the approach. While being responsive to evolving technologies, the goal is to foster mutual understanding among students and instructors while developing and maintaining the graduation projects. However, it is students and instructors responsibility to select a software process model and perform the activities and tasks suitable for their production.

The findings of this study have a number of important implications for future practice. Taken together, our results will be important to explore the potentials of this approach for other universities working on graduation project planning and improvement. The evidence from this study suggests that an iterative approach, designed with an incremental delivery structure, has allowed students to produce better quality work by drawing on the project development activities from the early parts of the semester. Suggested improvements have allowed us to receive positive feedback from various student project teams and ultimately they have made a significant contribution to achieving the graduate student qualifications expected of graduates of the engineering faculty. A significant number of graduation projects that were conducted using this process were entitled to benefit from the science fellowships and grant programs of TÜBİTAK—the Scientific and Technological Research Council of Turkey.
